# Sport Hunting, Predator Control and Conservation of Large Carnivores

**DOI:** 10.1371/journal.pone.0005941

**Published:** 2009-06-17

**Authors:** Craig Packer, Margaret Kosmala, Hilary S. Cooley, Henry Brink, Lilian Pintea, David Garshelis, Gianetta Purchase, Megan Strauss, Alexandra Swanson, Guy Balme, Luke Hunter, Kristin Nowell

**Affiliations:** 1 Department of Ecology, Evolution and Behavior, University of Minnesota, Saint Paul, Minnesota, United States of America; 2 Wildlife Demographics, Logan, Utah, United States of America; 3 Durrell Institute of Conservation and Ecology, Kent University, Canterbury, Kent, United Kingdom; 4 The Jane Goodall Institute, Arlington, Virginia, United States of America; 5 Minnesota Department of Natural Resources, Grand Rapids, Minnesota, United States of America; 6 The Zambesi Society, Bulawayo, Zimbabwe; 7 Panthera, New York, New York, United States of America; 8 Cat Action Treasury, Cape Neddick, Maine, United States of America; University of Pretoria, South Africa

## Abstract

Sport hunting has provided important economic incentives for conserving large predators since the early 1970's, but wildlife managers also face substantial pressure to reduce depredation. Sport hunting is an inherently risky strategy for controlling predators as carnivore populations are difficult to monitor and some species show a propensity for infanticide that is exacerbated by removing adult males. Simulation models predict population declines from even moderate levels of hunting in infanticidal species, and harvest data suggest that African countries and U.S. states with the highest intensity of sport hunting have shown the steepest population declines in African lions and cougars over the past 25 yrs. Similar effects in African leopards may have been masked by mesopredator release owing to declines in sympatric lion populations, whereas there is no evidence of overhunting in non-infanticidal populations of American black bears. Effective conservation of these animals will require new harvest strategies and improved monitoring to counter demands for predator control by livestock producers and local communities.

## Introduction

**Figure 1:**
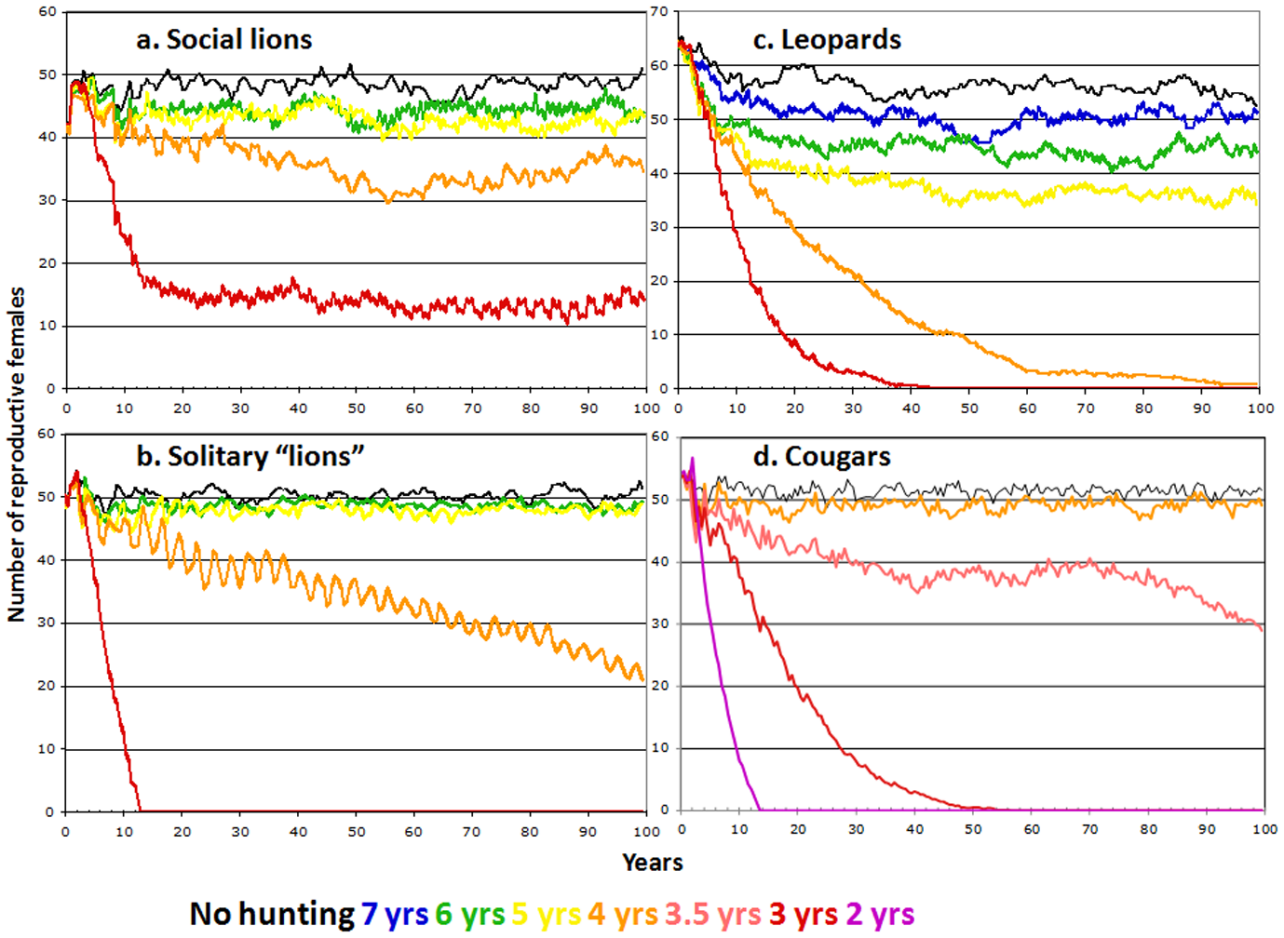
Average number of adult females in population simulations where all eligible males are removed during a 6-mo hunting season each year for 100 yrs. Colors indicate outcomes for different age minima for trophy males; each line indicates average from 20 runs. A. Population changes for “social lions” follow the assumptions and demographic variables in ref. [1] except to restrict hunting to 6-mo seasons and to incorporate additional details of dispersal, survival and reproduction [44]–[46]. B. Population changes for a hypothetical lion population where males and females are solitary and each territorial male controls one female. C. Population changes for leopards based on long-term data from Phinda Private Game Reserve [33], [47] and other sources [37], [48]. D. Population changes for cougars based on demographic data from refs. [27], [49]–[53].

Management agencies typically skew harvests toward males in order to protect adult females. However, in species with extensive paternal investment such as African lions (*Panthera leo*), trophy hunting can increase the rate of male replacement (and associated infanticide) to the point of reducing population size unless offtakes are restricted to males old enough to have reared their first cohort of dependent offspring (≥5–6 yrs of age) [1]–[3]. Solitary felids have none of the “safety nets” provided by the cooperative cub rearing strategies of African lions [4]–[5], and Fig. 1ab illustrates the greater vulnerability of solitary species by examining the effects of trophy hunting on a hypothetical population of “solitary lions” while leaving other demographic parameters from ref. [1] unchanged (Supporting Information Table S1, also see ref. [6]). Leopards (*Panthera pardus*) may be more sensitive to sport hunting than solitary lions (with a safe minimum age of 6–7 yrs of age, Fig. 1c), whereas cougar (*Felis concolor*) males can be safely harvested as young as 4 yrs of age (Fig. 1d).

We tested whether infanticidal species are vulnerable to over-hunting by focusing on four large carnivore species with sizable markets for sport-hunted trophies, comparing three infanticidal felids (lions, cougars and leopards) to American black bears (*Ursus americanus*). We used black bears as a control case because males do not kill cubs in order to increase mating opportunities (sexually-selected infanticide – SSI), so rates of infanticide are not increased by male-biased trophy hunting; in fact, among ursids, SSI has been documented in only one population of European brown bears (*U. arctos*) [7]–[9].

We extracted data from the UNEP World Conservation Monitoring Centre (WCMC) CITES trade database (See Materials and Methods). Data on total trophy harvests of lions and leopards are not available, so we used CITES-reported exports, which in cougars and black bears were highly correlated with domestic sport-hunting totals (Supporting Information Fig. S1); likewise CITES-reported trade in Tanzania's lion trophies showed a close match between imports and exports. Given sustained market demand, harvest trends should provide a reasonable proxy of population trends since sport hunters use intensive methods such as baits and hounds to locate these animals, and quotas on annual offtakes are either too high to limit harvests or (for black bears) reflect the management agency's perception of population trend [10].

## Results

**Figure 2:**
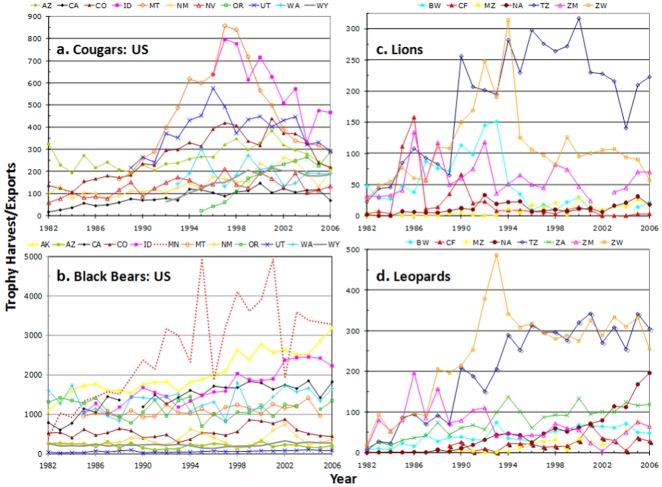
Domestic offtakes of a) cougars and b) black bears and CITES-reported trophy exports of c) lions and d) leopards. For US states: AK = Alaska, AZ = Arizona, CA = California, CO = Colorado, ID = Idaho, MN = Minnesota, MT = Montana, NM = New Mexico, NV = Nevada, OR = Oregon, UT = Utah, WA = Washington, WY = Wyoming. For CITES data: BW = Botswana, CF = Central African Republic, MZ = Mozambique, NA = Namibia, TZ = Tanzania, ZM = Zambia, ZW = Zimbabwe.

Fig. 2 shows the annual CITES exports for lions and leopards and US offtakes of cougars and black bears (See Materials and Methods). The reported number of trophies increased rapidly across all four species as markets grew during the 1980's and 1990's [11]–[12]. Offtakes have continued to increase for black bears, reflecting the sustained growth of bear populations throughout North America [13]. Leopard offtakes reached an asymptote in most countries, except for declines in Zambia in the 1980's and Zimbabwe in the 1990's and a recent CITES-granted increase to Namibia. In contrast, lion offtakes peaked then fell sharply in the 1980's and 1990's in Botswana, Central African Republic, Namibia, Tanzania, Zambia and Zimbabwe. Cougar offtakes showed similar peaks and declines in the 1990's in Arizona, Colorado, Idaho, Montana and Utah (Fig. 2).

The downward harvest trends for lions and cougars (highlighted in Supporting Information Fig. S2) most likely reflected declining population sizes: success rates (as measured by harvest/quota) have fallen for both cougars and lions (Supporting Information Fig. S3). Demand for lion trophies (as measured by total imports from across Africa) has grown in the US and held stable in the EU since the mid-1990s, sustained in recent years by imports of trophies of captive lions from South Africa [12], [14] (Supporting Information Fig. S3). Several countries instituted temporary bans on lion trophy hunting (Botswana in 2001–2004, Zambia in 2000–2001 and western Zimbabwe in 2005–2008) or banned female lions from quota (Zimbabwe, starting in 2005), but these measures were implemented well after the major decline in lion offtake in each country. The harvest trends are also consistent with recent surveys suggesting a 30% continent-wide population decline in African lions [15] and declining cougar populations in several US states [16]–[17]. Conversely, black bear populations appear to be increasing across their range [13], even in states where cougar populations have declined (Fig. 2). Although not apparent from most hunting offtakes, leopards have undergone an estimated range decline of 35% in Africa [18] and were recently listed as Near Threatened by IUCN due to habitat loss, prey depletion, illegal skin trade and problem animal conflicts [19].

**Figure 3:**
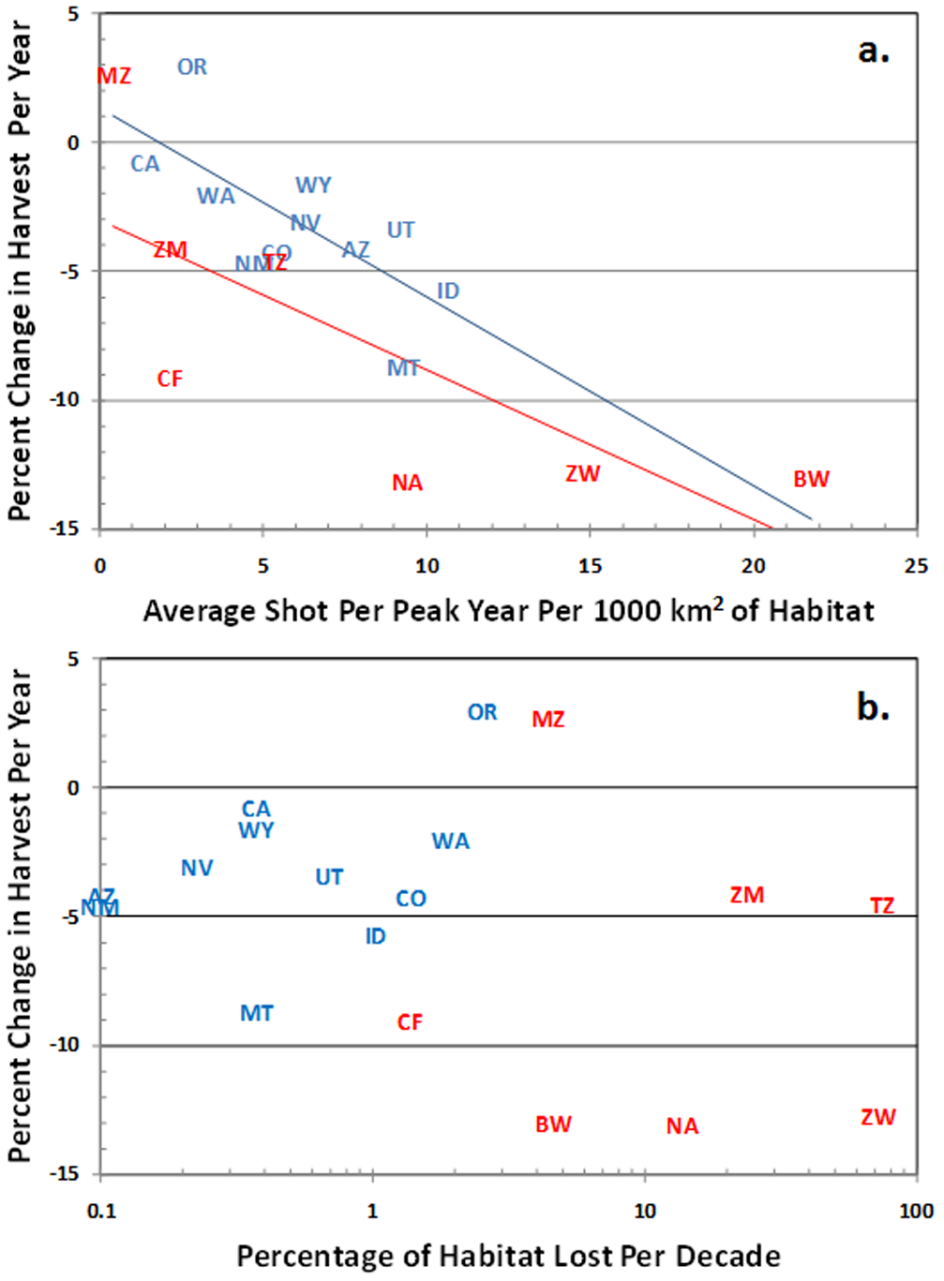
Recent trends in cougar offtakes (blue) and lion offtakes (red) as functions of a) harvest intensity and b) habitat loss. Jurisdictions with the highest harvest intensity showed the greatest decline in cougar offtakes (r^2^ = 0.5151, P = 0.0129) and lion offtakes (r^2^ = 0.5796, P = 0.0468). Habitat loss is plotted on a log scale to allow comparison between the African countries and the US states.

Trophy hunting is likely to have contributed to the declines in lion and cougar populations in many areas. Over the past 25 yrs, the steepest declines in cougar and lion harvests occurred in jurisdictions with the highest harvest intensities (Fig. 3a). Similarly, hunting blocks with the highest lion offtakes per 1000 km^2^ in Tanzania's Selous Game Reserve showed the steepest declines between 1996 and 2008 (r^2^ = 0.26, n = 45 blocks, P = 0.0004). The Selous is the largest uninhabited hunting area in Africa (55,000 km^2^) and has long been the premier destination for lion trophies. Across jurisdictions, declining harvests were unrelated to habitat loss for either lions or cougars (Fig. 3b) or to snow conditions for cougars. We modified our population simulation models to estimate impacts of sport hunting in a changing environment and found that habitat loss only imposes an additive effect on the impact of trophy hunting (Supporting Information Fig. S4). Note that habitat loss in many African nations has been so extensive (Fig. 3b) that lion offtakes have failed to recover for 10–20 yrs following the peak harvest years except in Namibia.

Although trophy hunting of lions and cougars is often portrayed as an economic strategy for increasing support for carnivore conservation, local communities often seek extirpation of problem animals [15], [20]–[22]. Thus, sport hunting quotas may sometimes reflect pressures to control carnivores rather than to conserve them. Across Africa, countries with the highest intensity of lion offtake also had the highest number of livestock units per million hectares of arable land (P = 0.047, n = 7). In the US, Oregon announced plans in 2006 to reduce its cougar population by 40% to decrease depredation on livestock, pets and game mammals [23], Washington altered its cougar quotas in response to human-wildlife conflicts in the 1990s–2000s, and recent offtakes have exceeded government-sanctioned eradication programs in several states. For example, Utah's sport-hunting cougar harvests averaged 500/yr in 1995-7 compared to peak culls of 150/yr in 1946–1949 [24], and Montana sport hunters harvested 800/yr in 1997–1999 vs. 140/yr in the peak “bounty” years of 1908-11 [25]. Likewise, South Africa exported 120 leopard trophies per year in 2004–2006, similar to the cull of 133 leopards per year in Cape Province (which covered most of the country) during 1920–1922 [26].

**Figure 4:**
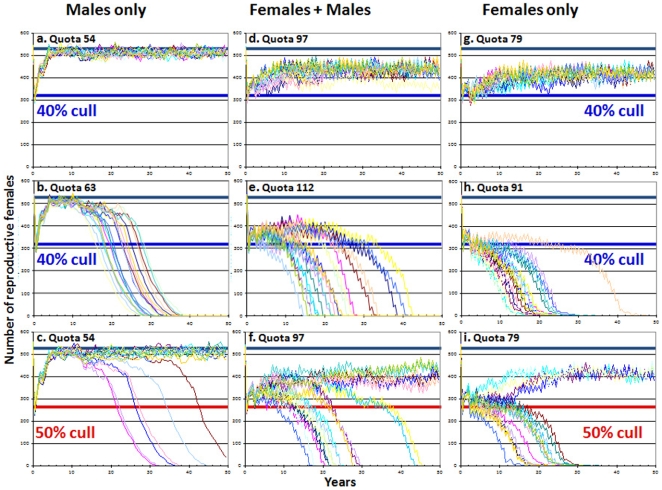
Simulated cougar populations subjected to an initial cull followed by fixed offtakes for 50 yrs. The initial cull is either 40% (top and middle rows) or 50% (bottom row), and the subsequent harvests are either the maximum offtake that incurred no extinctions in 20 runs following a 40% cull (top and bottom rows) or the minimum that produced 20 extinctions in 20 runs following a 40% cull (middle row). In the absence of sport hunting, the stable population size in these simulations is 527 reproductive females (indicated by the heavy black line in each graph); a 40% reduction in population size is indicated by blue lines, a 50% reduction by red lines. Each column represents a different harvest strategy: male only (left column), males and females (middle) and female only (right). Demographic parameters are set as in Fig. 1; quotas allow offtake of animals as young as 2 yrs; each graph shows outputs from 20 runs.

Fig. 4 shows the potential consequences of coupling a 40% cull of cougars with intensive sport hunting if the control program only targets males (reflecting traditional trophy hunting), removes males and females in proportion to their abundance, or only removes adult females. Fig. 4adg show population trends for the maximum fixed offtakes that never resulted in population extinctions during 20 simulations, whereas Fig. 4beh show the minimum fixed harvests that caused extinction in all 20 runs (often within 10 yrs of an initial decline). Fig. 4cfi show the consequences of applying the maximum “safe” offtakes if the population were inadvertently culled by 50% because of inaccurate population estimates. Consistent with population viability analyses [27]–[28], a female-only harvest comes closest to maintaining a persistent population reduction; a mixed male-female strategy allows the largest number of trophies to be harvested; a male-only harvest never maintains a 40% reduction in population size and has the smallest margin of error (male-only harvests can have catastrophic effects even in non-infanticidal species [29]).

**Figure 5:**
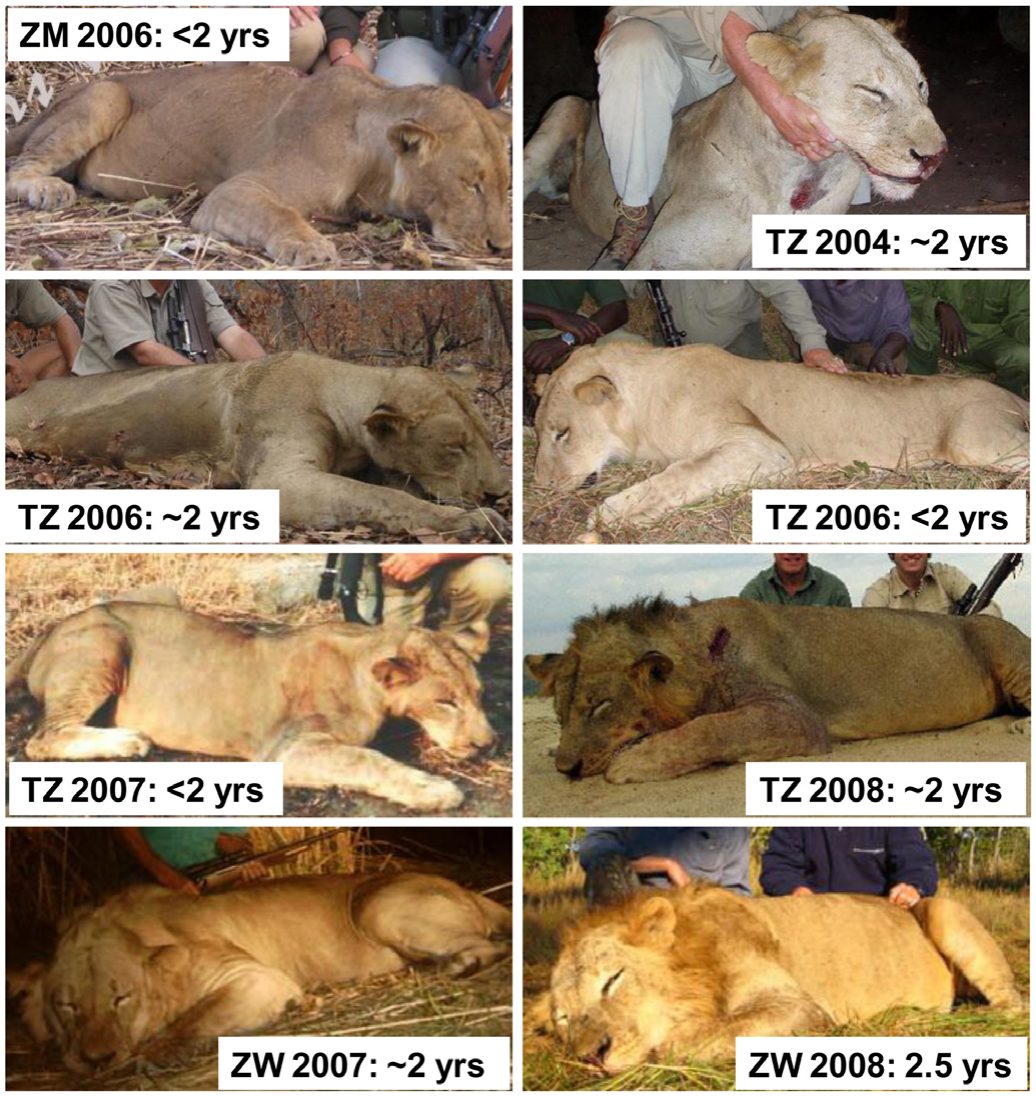
Sample of under-aged male African lions shot by sport hunters in various countries from 2004–2008.

These simulations assume a fixed harvest whereas many wildlife agencies reduce their quotas in response to lowered offtakes (Supporting Information Fig. S3 also see ref. [30]). However, offtakes may often be maintained at constant levels through compensatory increases in hunting effort, running the risk of an “anthropogenic Allee effect” [31]–[32]. Hunters in Zambia, Zimbabwe and Tanzania maintain their lion harvests by shooting males as young as 2 yrs of age (Fig. 5). In Zimbabwe, high lion offtakes were sustained from 1995 until 2005 by allowing females on quota [3], and the duration of lion safaris increased by nearly 18% from 1997 to 2001 (Supporting Information Fig. S3). Similarly, hounds have been used to hunt leopards in Zimbabwe since 2001, potentially masking a continued population decline.

## Discussion

Mortality from state-sanctioned and illegal predator control likely contributed to the overall population declines of cougars and lions; while leopards are also killed as pests, the leopard's CITES Appendix I status requires international approval for national export quotas, potentially providing safeguards against overharvest. However, leopard exports have declined in some countries, quotas have risen in others, and concerns have been raised over the level of problem animal offtakes and the management of leopard hunting practices [33]–[35]. Further, leopard populations in many areas may have been “released” [36] by large scale declines in lion numbers: lions inflict considerable mortality on leopards [37]; consequently, hunting blocks in Tanzania's Selous Game Reserve with the highest lion harvest intensities showed the largest *increases* in leopard harvests (P = 0.0059 after controlling for declines in lion offtakes, n = 45 blocks). Thus the full impact of current trophy hunting practices on leopards may not be fully apparent for several more years.

Harvest policies for infanticidal species such as lions, cougars and leopards that relied on “constant proportion” or “fixed escapement” could help protect populations but require accurate information on population size and recruitment rates, which are virtually impossible to collect; a harvest strategy of “constant effort” can more easily be achieved by measuring catch rates and regulating client days [38]–[40]. Hunting efficiency could be reduced by banning or limiting the use of baits and hounds, but the absence of direct oversight in remote hunting areas would make enforcement difficult. Alternatively, the age-minimum harvest strategies illustrated in Fig. 1 could be implemented without risk of over-hunting, assuming that ages can be reliably estimated before the animals are shot [41] rather than afterwards [42]. Unsustainable levels of trophy hunting of lions and cougars appear to be driven by conflicts with humans and livestock: the intensity of lion hunting was highest in countries with the most intensive cattle production, and wildlife managers are under similar pressure from US ranchers to raise cougar offtakes. Thus an even more fundamental challenge for carnivore conservation will be to build community tolerance for predators by reducing the need for retaliatory predator control and by improving benefit sharing from well managed trophy hunting [15].

## Materials and Methods

We analyzed trophy exports (http://www.unep-wcmc.org/citestrade/) by using the term “trophy” and restricting the analysis to countries that exported at least 25 trophies of a particular species for at least 2 yrs from 1982 to 2006 (excluding captive-bred lion trophies from South Africa). Other types of exports (skins) were also analyzed for lions, since non-standard terms are sometimes used by reporting countries [43], but these did not alter overall export trends. Data on Tanzanian hunting quotas were provided by the CITES office at the Division of Wildlife headquarters in Dar es Salaam; data on duration of hunting safaris in Zimbabwe were from the head office of Parks and Wildlife Management Authority in Harare.

Offtake data for black bears and cougars were provided by the Alaska Dept. of Fish & Game, Arizona Game & Fish Dept., California Dept. of Fish & Game, Colorado Division of Wildlife, Idaho Fish & Game, Minnesota Dept. of Natural Resources, Montana Fish, Wildlife & Parks, New Mexico Game & Fish, Nevada Dept. of Wildlife, Oregon Dept. of Fish & Wildlife, Utah Division of Wildlife Resources, Washington Dept. of Fish & Wildlife, and Wyoming Game & Fish. Note that all cougar offtakes in California are due to predator control.

“Harvest intensity” is the average harvest of the three peak offtake years divided by the extent of habitat in that state/country. Regression coefficients were calculated across the time period beginning with the earliest of the three peak harvests and ending in 2006 for cougars or the last of the three lowest subsequent harvest years for lions (Supporting Information Fig. S3); percent change is the regression coefficient divided by the peak harvest. Limited lion and leopard offtake data were available from 1996–2008 in Tanzania's hunting blocks; trends were only calculated for blocks reporting ≥5 yrs of activity.

Cougar habitat is forest cover taken from the National Land Cover Database (NLCD) www.mrlc.gov/changeproduct.php; lion habitat is the extent of GLOBCOVER land classification categories 42, 50, 60, 70, 90, 100, 110, 120, 130, 134, 135, 136, 160, 161, 162, 170, 180, 182, 183, 185, 186 and 187 in each country, see http://postel.mediasfrance.org/en/DOWNLOAD/Biogeophysical-Products/. Habitat loss is based on change in forest cover in the US 1990–2000 and in woodland/forest habitat in Africa 1990–2005 from FAO Global Forest Resources Assessment 2005, http://www.fao.org/forestry/32185/en/. Snow conditions for cougars are taken from http://www.wrcc.dri.edu/Climsum.html and African livestock production is taken from http://www.fao.org/es/ess/yearbook/vol_1_1/pdf/b02.pdf, using production levels from years of peak lion offtake in each country.

## Supporting Information

Figure S1 The number of CITES-reported exports of a) cougar trophies and b) black bear trophies from the US were highest in years when the most animals were harvested domestically in the western states (P<0.001 for each species).
(0.69 MB EPS)

Figure S2 Trendlines for the population declines of a) cougars and b) lions. Individual states with statistically significant declines in cougar offtakes: MT, ID, AZ, UT and CO; individual countries with significant declines in lion offtakes: BW, TZ and ZW.
(1.08 MB EPS)

Figure S3 Quotas, offtakes and catch rates each year since the peak harvests for cougars in Colorado, Montana and Utah and lions in Tanzania and Botswana; duration of lion hunts in Zimbabwe. Catch rates are (offtakes/quotas). Catch rates have generally declined because offtakes have fallen more quickly than quotas. Catch rates briefly improved in Utah and Botswana when quotas were adjusted downwards, but subsequently resumed an overall decline; Montana's adjustments in quotas are too recent to evaluate. For Zimbabwe, vertical lines indicate standard errors; numbers are sample sizes; duration of lion hunts became significantly longer between 1997 and 2001 (P<0.01). No other data are available on quotas or hunt durations from these or other countries/states. The bottom graphs show that declines in lion trophy exports are unlikely to reflect declining market demand; imports of lion trophies have increased, especially in recent years for captive-bred or “canned” lion trophies for South Africa. The declines in trophy exports are also unlikely to be caused by irregular reporting; adding additional exports of skins from Botswana, Tanzania and Zimbabwe would not significantly change the pattern of decline.
(1.38 MB EPS)

Figure S4 Simulated impacts of trophy hunting in cougars for varying degrees of habitat loss. Solid lines are the same as in Fig. 1: all available males above the age minimum are harvested each year and available habitat remains unchanged over 100 yrs. Dashed lines show population sizes with the same harvest strategies but with 20% habitat loss in 100 yrs; dotted lines represent outputs with 40% habitat loss.
(1.49 MB EPS)

Table S1 (0.03 MB DOC)
