# Supplementary material for: Sport Hunting, Predator Control and Conservation of Large Carnivores
Source: PLoS One. 2009 Jun 17;4(6):e5941. doi: 10.1371/journal.pone.0005941 (PMC2691955; doi:10.1371/journal.pone.0005941)
Supplement: Table S1 — (0.03 MB DOC) [file pone.0005941.s005.doc]

**Simulation Models**

Details of the individually based, spatially explicit simulation model have been described previously [1-3]. The lion model consists of a network of linked hexagonal territories; individual females and their dependent offspring belong to a single territory while adult males form coalitions that can span multiple territories. Every six-month time step, each lion either advances in age or dies; subadult females either emigrate as a cohort or join their natal pride (according to the current number of adult females in the pride); adult females give birth depending on the age and survival of their prior offspring; males challenge each other for control of territories.

We modified the original lion model for solitary species as follows: subadult females must always disperse singly and can neither remain in the natal territory nor disperse as a cohort with their sisters; subadult males are unable to form coalitions and must disperse singly. For leopards and cougars, input parameter values reflect the biology and population dynamics of each species (Table S1). Except for subadult dispersal, the parameter values for “solitary lions” are identical to social lions.

To allow comparable numbers of reproductive females in Fig. 1, we use 10 territories for social lions and 52 territories for solitary lions, leopards, and cougars; Fig. S-4 also uses 52 cougar territories. Fig. 4 covers 529 cougar territories, yielding an initial starting population of 1899 individuals. All territory networks are as convex as possible.

We further modified the original model to harvest females and to hunt both males and females. Previous analyses [2,3] allowed trophy offtakes during every time step (twice per year); here we restricted hunting to every other time step (once per year) to better mimic seasonal hunting. To simulate the impact of a population cull followed by trophy hunting, reproductive individuals are randomly chosen until a predetermined percentage has been removed; hunting then proceeds with annual quotas determining offtake.

To examine the effects of habitat loss, we specify the percentage of territories to be deleted over a given time period. In each time step, the model removes territories so as to produce a linear rate of loss; all resident cubs die whereas subadult and adult females can move to empty adjacent territories but cannot reproduce if the territory is occupied. If the adult male only occupied the deleted territory, he becomes nomadic. We explored two scenarios of habitat loss. First, to mimic human encroachment, territories were only removed from the edge of the territory network. Second, to simulate habitat fragmentation, territories were eliminated at random. The two alternatives yielded similar results.

1. Quading HS, Starfield AM (2002) Exploiting object-oriented programming structures in the quest for an individual-based lion population model with an attractive user interface. South African Journal of Science 98: 449-454.

2. Whitman K, Starfield AM, Quadling HS, Packer C (2004) Sustainable trophy hunting of African lions. Nature 428: 175-178.

3. Whitman KL, Starfield AM, Quading H, Packer C (2007) Modeling the Effects of Trophy Selection and Environmental Disturbance on a Simulated Population of African Lions. Conservation Biology 21: 591-601.

| **TABLE S1.** | **Lions** |  | **ref** | **Leopards** | | **ref** | **Cougars** | | ref |
| --- | --- | --- | --- | --- | --- | --- | --- | --- | --- |
| **Age-specific survival per 6-m** |  | **age range** | |  | **age range** | |  | **age range** | |
| Cubs | 0.770 | 0-6 m | a | 0.768 | 0-6 m | e,f,g | 0.742 | 0-1 y | h |
|  | 0.830 | 6-12 m | a | 0.768 | 6-18 m | e,f,g |  |  |  |
|  | 0.870 | 1-2 y | a |  |  |  |  |  |  |
| Subadult male | 0.930 | 2-4 y | b | 0.943 | 1.5-3.5 y | e,g | 0.774 | 1-3 y | h,i |
| Subadult female | 0.986 | 2-4 y | a | 0.964 | 1.5-3.5 y | e,g | 0.933 | 1-3 y | h,i |
| Adult resident male | 0.950 | 4+ y | b | 0.856 | 3.5+ y | e,g | 0.824 | 3+ y | h,j |
| Adult nomadic male | 0.900 | 4+ y | b | 0.856 | 3.5+ y | * | 0.824 | 3+ y | * |
| Adult female | 0.986 | 4+ y | a | 0.933 | 3.5+ y | e,g | 0.933 | 3+ y | j,k |
| Adult second-time nomadic male | 0.400 | 4+ y | a | 0.856 | 3.5+ y | * | 0.824 | 3+ y | * |
| Adult homeless female | 0.500 | 4+ y | b | 0.933 | 3.5+ y | * | 0.933 | 3+ y | * |
| Orphaned cub | 0.500 | 0-2 y | a | 0.000 | 0-18 m | ** | 0.000 | 0-1 y | ** |
|  |  |  |  |  |  |  |  |  |  |
| Max. reproductive age | 13 | y | a | 15 | y | f | 13 | y |  |
| Min. female reproductive age | 3 | y | b | 3 | y | e,f | 2 | y | i |
| Min. male reproductive age | 2.5 | y | b | 2.5 | y | e | 2 | y | i |
|  |  |  |  |  |  |  |  |  |  |
| **Litter size probabilities** |  |  |  |  |  |  |  |  |  |
| 1 cub | 0.19 |  | c | 0.25 |  |  | 0.05 |  | i,l |
| 2 cubs | 0.35 |  | c | 0.25 |  |  | 0.27 |  | i,l |
| 3 cubs | 0.33 |  | c | 0.25 |  |  | 0.49 |  | i,l |
| 4 cubs | 0.13 |  | c | 0.25 |  |  | 0.19 |  | i,l |
|  |  |  |  |  |  |  |  |  |  |
| Prob. each cub is male | 0.50 |  | a | 0.50 |  |  | 0.50 |  |  |
| Prob. single cub abandoned | 0.70 |  | a | 0.00 | *** |  | 0.00 | *** |  |
|  |  |  |  |  |  |  |  |  |  |
| **Prob. subadult females disperse to unoccupied territories** | | | | |  |  |  |  |  |
| Adjacent territory | 1.00 |  | a | 1.00 |  |  | 1.00 |  |  |
| 2 territories away | 0.80 |  | d | 0.80 |  |  | 0.80 |  |  |
| 3 territories away | 0.30 |  | d | 0.30 |  |  | 0.30 |  |  |
| 4 territories away | 0.15 |  | d | 0.15 |  |  | 0.15 |  |  |
| 5 territories away | 0.10 |  | d | 0.10 |  |  | 0.10 |  |  |
| ≥6 territories away | 0.00 |  | d | 0.00 |  |  | 0.00 |  |  |
|  |  |  |  |  |  |  |  |  |  |
| **Male movement** |  |  |  |  |  |  |  |  |  |
| # territories nomads move in 6 m | 3 |  | d | 5 |  | e | 7 |  | m |
| # territories subadults visit in 6 m | 1 |  | d | 2 |  | e | 2 |  |  |
| Max. # of territories held by 1 male | 1 |  | a | 6 |  | e,g | 3 |  | i,k |
| Max. # of territories held by ≥2 males | 3 |  | a |  |  |  |  |  |  |
| Prob. lone nomad joins lone resident | 0.50 |  | a | 0.00 | **** |  | 0.00 | **** |  |
| Prob. 2 lone nomads join up | 1.00 |  | a | 0.00 | **** |  | 0.00 | **** |  |
| Prob. lone nomad joins 2-♂ coalition | 0.20 |  | a | 0.00 | **** |  | 0.00 | **** |  |
|  |  |  |  |  |  |  |  |  |  |
| **Competition and Takeovers** |  | **age range** | |  | **age range** | |  | **age range** | |
| Prob. cub survives takeover | 0.01 | 0-6 m | a | 0.01 | 0-6 m |  | 0.05 | 0-1 y |  |
|  | 0.25 | 6-12 m | a | 0.40 | 6-18 m |  |  |  |  |
|  | 0.65 | 1-2 y | a |  |  |  |  |  |  |
| Prob. defending mother survives | 0.95 |  | b | 0.95 |  |  | 0.95 |  |  |
| Prob. successful ♂ survives | 0.97 |  | a | 0.97 |  |  | 0.97 |  |  |
| Prob. unsuccessful defending ♂ lives | 0.40 |  | a | 0.40 |  |  | 0.40 |  |  |
| Prob. unsuccessful attacking ♂ lives | 0.50 |  | a | 0.50 |  |  | 0.50 |  |  |
|  |  |  |  |  |  |  |  |  |  |
| * resident same as non-resident | a = Whitman et al. 2004 | | | | g = Balme 2009 | | |  |  |
| ** orphans of solitaries never survive | b = Packer et al. 1988 | | | | h = Ruth et al. 2008 | | | |  |
| *** lone cubs not abandoned | c = Packer & Pusey 1995 | | | | i = Logan & Sweanor 2001 | | | |  |
| **** males always solitary | d = Craft et al. 2009 | | |  | j = Maehr & Moore 1992 | | | |  |
|  | e = Bailey 1993 | |  |  | k = Cooley 2008 | | | |  |
|  | f = Martin & de Meulenaer 1988 | |  |  | l = Lambert et al 2006 m = Sweanor et al 2000 | | | |  |

YELLOW: Updated parameter values for lions:

1) Male survival more accurately reflects background mortality

2) Observed distribution of litter sizes

3) Nomadic males now move 3 territories

BLUE: Additional aspects to original lion model:

1) Homeless females now survive, though at a low rate

2) The age of reproductive maturity is treated as a parameter

3) Subadult females can disperse >2 territories away

4) Mothers may die while defending their cubs

PURPLE: Cougar and leopard parameters either assigned "default" values or values from the other species:

1) Maximum age for cougars: same as lions.

2) Litter sizes for leopards: 25%-25%-25%-25% for 1, 2, 3 or 4 cubs

3) Sex ratio at birth assumed in all cases to be 50-50.

4) Subadult female dispersal for leopards and cougars: same as lions.

5) Mobility of subadult male cougars: same as leopards

6) Survival of cubs, defending mothers & attacking/defending successful/unsuccessful ♂ leopards/cougars: as lions.
